# Atherosclerosis risk factor management - what's new for the neurologist?

**DOI:** 10.1590/0004-282X-ANP-2022-S102

**Published:** 2022-08-12

**Authors:** Luciana Dornfeld Bichuette, Marcos Pita Lottenberg, Francisco Akira Malta Cardozo, Daniela Calderaro

**Affiliations:** 1 Universidade de São Paulo, Faculdade de Medicina, Hospital das Clínicas, Instituto do Coração, São Paulo SP, Brazil.

**Keywords:** Stroke, Atherosclerosis, Risk Factors, Acidente Vascular Cerebral, Aterosclerose, Fatores de Risco.

## Abstract

Stroke is the second leading cause of death worldwide and the vast majority can be attributed to modifiable risk factors, mainly behavioral and metabolic. The top six risk factors responsible for cardiovascular mortality in Brazil in 2019 were high systolic blood pressure, inadequate dietary exposure, high body mass index, high LDL cholesterol, high fasting blood glucose levels, and tobacco. We intend to discuss in this paper the evidence and recommendations in the approach of three essential risk factors for patients with a history of stroke: dyslipidemia, hypertension and diabetes.

## RESUMO

O acidente vascular cerebral (AVC) é a segunda causa de morte no mundo e, em grande parte, pode ser atribuído a fatores de risco modificáveis, principalmente comportamentais e metabólicos. Os seis principais fatores de risco responsáveis pela mortalidade cardiovascular no Brasil em 2019 foram pressão arterial sistólica elevada, exposição alimentar inadequada, índice de massa corporal elevado, LDL -colesterol elevado, níveis alterados de glicemia e tabagismo. Pretendemos discutir neste artigo as evidências e recomendações na abordagem de três fatores de risco essenciais para pacientes com histórico de AVC: dislipidemia, hipertensão e diabetes.

## INTRODUCTION

Stroke is the second leading cause of death worldwide and the third leading cause of disability^1^. In the United States, the proportion of cases of ischemic stroke, intracerebral hemorrhage, and subarachnoid hemorrhage is 87%, 10%, and 3%, respectively. These rates appear to be similar globally, with a trend towards a higher frequency of hemorrhagic forms of stroke in developed countries compared to developing countries^2^.

In recent years, studies have shown a reduction in recurrent stroke and transient ischemic attacks (TIAs) rates, as prevention strategies have been implemented and improved^3^. A meta-analysis of randomized controlled trials published from 1960 to 2009 showed a reduction in annual stroke recurrence, from 8.7% in the 1960s to 5% in the 2000s. This reduction was driven mainly by better blood pressure control and antiplatelet therapy^4^.

The Global Burden of Disease (GBD) Study showed that approximately 90% of strokes could be attributed to modifiable risk factors, including behavioral factors such as smoking, poor diet, sedentary lifestyle, and metabolic factors such as high blood pressure, blood glucose, and cholesterol^5^. Analysis of the Brazilian data from the GBD Study confirms the global trend and reinforces the urge for better preventive strategies. The top six risk factors responsible for cardiovascular mortality in 2019 were high systolic blood pressure, inadequate dietary exposure, high body mass index, high LDL cholesterol, high fasting blood glucose levels, and tobacco^6^.

Although the combination of dietary modification, physical activity, use of aspirin, statins, and antihypertensive agents may result in a cumulative reduction in the relative risk of stroke recurrence by 80%^7^, the control of risk factors in patients with a history of stroke is not yet adequate. We intend to discuss the evidence and recommendations in the approach of three essential risk factors for patients with a history of stroke. The management of antithrombotic therapy will be discussed in a dedicated review in this paper.

## LIPID MANAGEMENT

Elevated low-density lipoprotein cholesterol (LDL-c) levels are associated with an increased risk of ischemic stroke, but this correlation is not evident in patients with hemorrhagic stroke. In addition, the incidence of atherothrombotic and lacunar strokes, but not of cardioembolic strokes, increases significantly with increased LDL-c levels^8^. High-density lipoprotein cholesterol (HDL-c) is inversely associated with the risk of ischemic stroke, especially atherothrombotic, and Sacco et al. found a protective effect when HDL-c is above 35 mg/dL^9^. Although the inverse epidemiological relationship between HDL-c levels and the incidence of cardiovascular disease is recognized, more recent studies have failed to demonstrate the clinical benefit of HDL-c increase through drug therapy^10^.

Given the association between serum LDL-c levels and ischemic stroke, the SPARCL (The Stroke Prevention by Aggressive Reduction in Cholesterol Levels[A1]) study compared the use of high-potency statins with placebo in patients who had had a stroke or TIA in the previous six months and had no known coronary disease. A 16% relative risk reduction for stroke was observed in the atorvastatin group, which is partly explained by the lower mean LDL-c level achieved in this group compared to placebo (LDL cholesterol, 72.9 ± 0.5 mg/dL in the atorvastatin group, compared with 128.5 ± 0.5 mg/dL in the placebo group). However, the anti-inflammatory effect of statins and reduction of endothelial dysfunction also contribute to this finding^11^. In a meta-analysis of 21 randomized studies, for every 1 mmol/L LDL (equivalent to 38.7mg/dL) of LDL-c reduction, there was a 21% reduction in the relative risk of ischemic stroke over five years of follow-up^12^.

Due to the residual risk of cardiovascular events despite high-potency statins therapy, additional treatments to reduce LDL-c have been implemented. More recently, the IMPROVE-IT study (Improved Reduction of Outcomes: Vytorin Efficacy International Trial) evaluated the use of Ezetimibe, a cholesterol absorption inhibitor, associated with statins in patients hospitalized for acute coronary syndrome, showing a significant reduction in the risk of ischemic stroke when compared with the use of statins alone, during a mean follow-up of six years^13^.

Proprotein convertase subtilisin/kexin type 9 (PCSK9) inhibitors, a class of monoclonal antibodies represented by evolocumab and alirocumab, are capable of reducing serum LDL-c levels by approximately 60%, proving to be a promising therapeutic class. The FOURIER study (Further Cardiovascular Outcomes Research with PCSK9 Inhibition in Subjects with Elevated Risk) evaluated the use of evolocumab versus placebo in patients with established atherosclerotic disease and LDL-c levels above 70 mg/dL in spite of statin therapy. The addition of evolocumab reduced LDL-c levels from a median of 92 mg/dL (2.4 mmol/L) to 30 mg/dL (0.78 mmol/L), with a consequent reduction in the rate of ischemic strokes, but no significant difference concerning hemorrhagic strokes. This finding was consistent in the subgroup of patients with previous ischemic stroke and those without a history of stroke^14^ ^,^ ^15^.

A meta-analysis of randomized controlled trials evaluated the risks and benefits of a more intensive reduction in LDL-c when compared to more permissive therapy. The final mean LDL-c level in the former group, adjusted for the size of the study, was 79 mg/dL, compared with 119 mg/dL in the latter group. More intensive treatment was associated with a decrease in the risk of recurrent stroke. The benefit was not statistically different between the various LDL-c reduction strategies, demonstrating that the serum cholesterol level reached is more important than the therapy itself^16^.

Current guidelines recommend that, in the presence of clinically manifested atherosclerotic disease in patients with previous ischemic stroke, LDL-c reductions of at least 50% should be considered. It is ideal to obtain LDL-c levels < 50 mg/dL. For primary prevention, LDL-c levels < 70 mg/dL are recommended in high-risk patients, < 100 mg/dL in those at intermediate risk, and < 130 mg/dL in those at low risk^17^. 

Lipoprotein(a) [Lp(a)] is a plasma lipoprotein consisting of a cholesterol-rich LDL particle with an apolipoprotein B100 molecule attached to an additional protein, apolipoprotein A (ApoA). It is synthesized by the liver, and more than 90% of its circulating levels are genetically determined, with little influence of diet or environmental factors^18^.

Several studies have suggested that high serum Lp(a) values constitute an independent risk factor for atherosclerotic disease through multiple distinct mechanisms. Lp(a) can be deposited on the vessel walls, and due to its LDL and ApoA particles' high propensity to oxidation, these particles become highly immunogenic and proinflammatory. The fact that Lp(a) is more subject to oxidation than LDL can facilitate its uptake by macrophages, which become foam cells, atherosclerosis precursors. In addition, ApoA is structurally similar to plasminogen, interfering with the fibrinolytic cascade and resulting in a high risk of thrombogenicity^19^.

Although the role of Lp(a) as a risk factor for stroke is not as well documented as for coronary artery disease, a meta-analysis of 41 studies showed an increased risk of ischemic stroke and large-artery atherosclerosis in individuals with elevated serum Lp(a) levels^20^.

The use of statins seems to be associated with an increase in Lp(a). Thus, although statin therapy results in cardiovascular protection, it is possible that patients who develop an increase in Lp(a) after starting statin therapy do not obtain the full benefit^21^. Consistent studies are needed to assess the role of the variation in Lp(a) plasma levels before and after initiation of statin therapy and its relationship with cardiovascular outcomes. 

While randomized trials evaluating therapies that reduce Lp(a) by 20 to 30%, such as niacin and cholesteryl ester transfer protein (CETP) inhibitors, did not provide evidence that Lp(a) reduction decreases the risk of cardiovascular events, recent data with PCSK9 inhibitors suggested a possible role in this regard^22^.

A post hoc analysis of the ODYSSEY Outcomes (Evaluation of Cardiovascular Outcomes After an Acute Coronary Syndrome During Treatment with Alirocumab) study evaluated the benefit of associating a PCSK9 inhibitor with optimized statin treatment in patients with LDL-c close to 70 mg/dL. The effects were evaluated according to the concomitant Lp(a) levels, and individuals with serum levels < 13.7 mg/dL had no reduction in cardiovascular events with the use of alirocumab, unlike those with at least slightly elevated levels^23^. These findings suggest that the reduction of Lp(a) with PCSK9 inhibitors seems to bring benefits in relation to cardiovascular outcomes.

Antisense oligonucleotides such as mipomersen and pilacarsen are small nucleotide sequences (DNA or RNA) that bind specifically to messenger RNA and inhibit protein synthesis. These molecules inhibit the hepatic synthesis of apolipoprotein B100, consequently reducing Lp(a) plasma concentrations. A randomized study evaluated their effect on Lp(a) in patients with established cardiovascular disease, demonstrating a dose-dependent decrease in Lp(a) serum levels, with a mean percentage reduction ranging from 35% to 80%^24^.

Lp(a) dosage should be considered at least once in a lifetime for every adult. The assessment of Lp(a) plasma levels may also be helpful in patients with a family history of premature cardiovascular disease and determine treatment strategies in individuals with a borderline estimated risk in the risk categories^25^. Clinical trials with antisense oligonucleotides targeted to inhibit Lp(a) synthesis are ongoing to test the hypothesis that Lp(a) reduction may reduce the risk of cardiovascular events, including stroke^26^.

## HYPERTENSION

Systemic arterial hypertension (SAH) is widely known to be the leading risk factor for stroke, and up to 50% of stroke events can be attributed to SAH. Clinical trials have documented that blood pressure control reduces stroke mortality in hypertensive patients and its recurrence in patients with a history of stroke or TIA. Thus, blood pressure management has become a primary and secondary prevention cornerstone.

## BLOOD PRESSURE AND PRIMARY PREVENTION

There is robust evidence that SAH screening and treatment prevents cardiovascular disease and reduces mortality^27^. The pressure target to be achieved is still the subject of much debate, especially in elderly patients. 

The SPRINT study (Systolic Blood Pressure Intervention Trial) demonstrated a reduction in cardiovascular outcomes in high-risk hypertensive individuals submitted to more stringent pressure control (target systolic blood pressure [SBP] < 120 mmHg) when compared to more permissive treatment (target SBP < 140 mmHg)^28^. Nevertheless, the former group presented higher rates of adverse events, with a higher risk of hypotension and acute kidney injury. Diabetic patients and patients with previous cerebrovascular events were not eligible for the SPRINT study. Current guidelines recommend a blood pressure target lower than 130/80 mmHg for most patients^29^.

For hypertensive patients over 65 years of age, the current recommendation is to maintain blood pressure below 140/90 mmHg. Nevertheless, the recently published STEP (Strategy of Blood Pressure Intervention in Elderly Hypertensive Patients) study, which involved hypertensive patients aged 60 to 80 years, showed a lower incidence of cardiovascular events, including stroke, in those undergoing intensive treatment (SBP target 110-130 mmHg) compared to standard treatment (SBP target 130-150 mmHg)^30^.

## BLOOD PRESSURE MANAGEMENT TO PREVENT STROKE RECURRENCE

There are gaps in the evidence for blood pressure management in the secondary prevention of stroke, and more evidence is needed. The BOSS study (Blood Pressure and Clinical Outcomes in TIA or Ischemic Stroke) evaluated the association between systolic blood pressure and clinical outcomes in patients with a history of ischemic stroke or TIA. It demonstrated a probable "J"-shaped curve effect, in which both SBP < 115 mmHg and ≥ 165 mmHg were related to more significant recurrence of stroke when compared to SBP values between 125 and 134 mmHg^31^.

A meta-analysis of four randomized controlled trials comparing the effect of standard versus intensive blood pressure control in stroke recurrence showed more significant benefit in the latter group^32^. Thus, current guidelines recommend a blood pressure target lower than 130/80 mmHg for most patients. The Optimal Blood Pressure for the prevenTIon of Major vAscuLar Events in Stroke Patients (OPTIMAL Stroke) is a Brazilian clinical trial designed to test whether a lower target systolic blood pressure (SBP <120mmHg) as compared to the currently recommended target for stroke patients (SBP < 140mmHg) will reduce the occurrence of major cardiovascular events. The trial is still ongoing, with an estimated enrollment of 7104 participants^33^. Diuretics, angiotensin-converting enzyme (ACE) inhibitors, and angiotensin receptor blockers (ARBs) have shown benefits in systematic reviews. Although there are limited data on the efficacy of calcium channel blockers in the secondary prevention of stroke, their use can be considered as an additional option since the magnitude of the reduction in blood pressure seems to be more critical than the antihypertensive agent used^34^.

Additional studies are needed to determine the optimal moment for lowering blood pressure after stroke. Therefore, such recommendations concern the outpatient treatment of patients with stable neurological status.

## DIABETES MELLITUS

Type 2 diabetes mellitus (T2DM) is a significant risk factor for macrovascular complications, including acute myocardial infarction and stroke, which account for approximately 80% of deaths in T2DM patients^35^. Findings from the Emerging Risk Factors Collaboration showed that the adjusted risk rates were 2.27 (1.95-2.65) for ischemic stroke and 1.56 (1.19-2.05) for hemorrhagic stroke^36^. The risk of stroke in patients with diabetes is even more pronounced for women (HR: 2.83 for women vs 2.16 for men) and younger patients (HR: 3.74 for age group 40 to 59 and 1.80 for patients older than 70)^36^. The increase in diabetes incidence, related to bad eating habits, obesity, and sedentary lifestyle, resulted in exponential cardiovascular morbidity.

The contemporary concept of diabetes treatment for patients with established, or those at high risk of, heart disease involves not only glycemic control but also the use of drugs with proven reduction in cardiovascular events. There is now robust evidence for two new classes of drugs: the sodium-glucose cotransporter 2 (SGLT2) inhibitors and the Glucagon-like peptide 1 (GLP-1) analogs. Their evidence in major cardiovascular events reduction is greater than evidence favoring metformin^37^. 

Sodium-glucose cotransporter 2 (SGLT2) inhibitors are a new class of oral hypoglycemic agents for the treatment of T2DM, their mechanism being the inhibition of glucose and sodium reabsorption from the proximal renal tubule, thereby reducing glucose levels in the blood. A meta-analysis involving five randomized controlled trials showed no significant effect of this drug class on stroke rates. Subgroup analysis indicated that SGLT2 inhibitors were not associated with fatal and non-fatal stroke, ischemic stroke, and TIA reductions. However, they were associated with a significant reduction in hemorrhagic stroke compared to placebo^38^. Despite the fact that stroke reduction has not been directly demonstrated with the use of SGLT-2 inhibitors, diabetic patients with atherosclerotic disease receive enormous benefit in the global reduction of cardiovascular events, notably reduction of morbidity from heart failure. In the EMPA-REG study, a significant reduction in overall mortality was attributed to empagliflozin^39^.

Glucagon-like peptide 1 (GLP-1) analogs are another class of oral hypoglycemic agents whose mechanism lies in increasing insulin secretion and reducing glucagon secretion in a glucose-dependent manner. A recent meta-analysis demonstrated a reduction in stroke with their use, and additional analyses also demonstrate a reduction in cardiovascular events in patients with a history of previous stroke^40^. GLP-1 analogs remain underused in clinical practice, in part due to the cost. However, given its benefits, its use should be prioritized in high-risk patients with a history of stroke.

Therefore, for diabetic patients with cardiovascular disease, namely previous ischemic stroke due to atherosclerosis, regardless of previous metformin therapy, there is an indication for prescription of GLP-1 analog or SGLT2 inhibitors. After the initial therapy, the goal of hemoglobin A1c (HbA1c) must be assessed and additional therapy prescribed accordingly^37^. 

The therapeutic approach of diabetes in patients with a previous episode of stroke or transient ischemic attack should be individualized, as literature still lacks good evidence regarding goals of HbA1c in this specific population. According to a couple of relatively old studies that compared intensive versus lenient approaches in a general population of diabetes, an aggressive strategy delays the progression of microvascular complications related to diabetes^41^ ^,^ ^42^. In this context, the 2021 Guideline for the Secondary Prevention of Ischemic Stroke recommends a goal of HbA1c below 7%, especially in those with low risk of hypoglycemia^34^.

**Figure 1.**
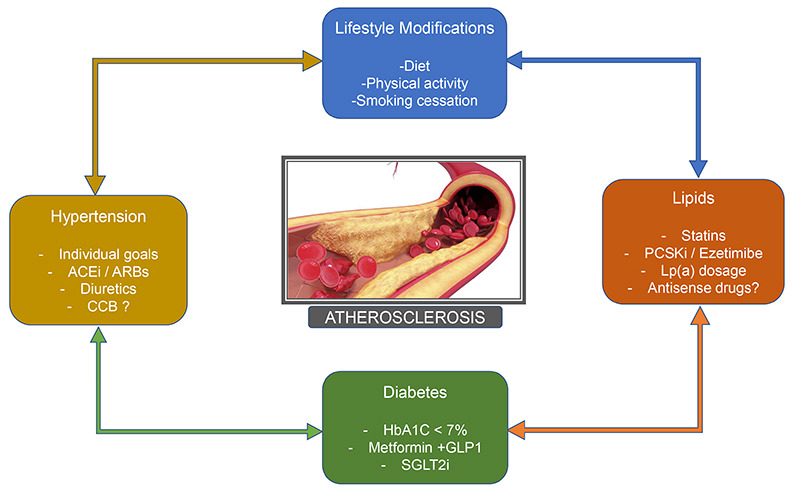
pharmacological and non-pharmacological measures for risk factors control.

ACEi: Angiotensin-Converting Enzyme inhibitors; ARBs: Angiotensin II Receptor Blockers; CCB: Calcium Channel Blockers; PCSKi: Proprotein convertase subtilisin/kexin type 9 inhibitors; Lp(a): Lipoprotein(a); HbA1C: Hemoglobin A1c; GLP1: Glucagon-like peptide 1 agonists; SGLT2i: Sodium-glucose cotransporter 2 inhibitors.

In conclusion, the assertive approach of risk factors for atherosclerosis is fundamental for the primary and secondary prevention of stroke (Figure 1). Non-pharmacological measures associated with pharmacological therapy can reduce cardiovascular morbidity and mortality in this scenario.
